# Nuclear imaging of inflammation: homing-associated molecules as targets

**DOI:** 10.1186/2191-219X-3-1

**Published:** 2013-01-03

**Authors:** Anu Autio, Sirpa Jalkanen, Anne Roivainen

**Affiliations:** 1 Turku PET Centre, University of Turku and Turku University Hospital, Kiinamyllynkatu 4-8, Turku, 20521, Finland; 2 MediCity Research Laboratory and Department of Medical Microbiology and Immunology, University of Turku, Turku, 20520, Finland; 3 Turku Center for Disease Modeling, University of Turku, Turku, 20520, Finland

**Keywords:** Inflammation, *In vivo* imaging, Positron emission tomography, Vascular adhesion protein-1

## Abstract

The golden standard in nuclear medicine imaging of inflammation is the use of autologous radiolabeled leukocytes. Although their diagnostic accuracy is precise, the preparation of the leukocytes is both laborious and potentially hazardous for laboratory personnel. Molecules involved in leukocyte migration (homing-associated molecules) could serve as targets for the development of imaging agents for inflammation. An excellent target would be a molecule that is absent or expressed at low levels in healthy tissues, but is present or upregulated at the sites of inflammation. In this paper, we will review the literature concerning the use of homing-associated molecules as imaging targets. We will especially concentrate on vascular adhesion protein-1 due to the promising results regarding its use as a target for the imaging of inflammation.

## Review

### Introduction

Noninvasive imaging of inflammation could be a highly valuable tool as it could help diagnosing many inflammatory conditions, such as osteomyelitis, rheumatoid arthritis, sarcoidosis, inflammatory bowel disease, and fever of unknown origin [1,2]. Even though there are several imaging agents used for the imaging of inflammation by nuclear medicine, none of them are specific for inflammation [2]. Specific imaging of inflammatory processes is a demanding task. Unspecific accumulation, as a result of an increased blood flow and enhanced vascular permeability, increases the signal in the target tissue which makes specific detection difficult. Positron emission tomography (PET) imaging of inflammation offers a noninvasive, quantitative approach to diagnosis, therapy planning, and monitoring and to drug development. Nevertheless, clinical applications of this method are currently limited by the lack of specific imaging agents to visualize molecular events *in vivo*. ^18^F]FDG is the most widely used PET imaging agent in cancer and inflammation imaging, but it localizes general inflammation or cancer and does not define these specifically.

### Nuclear imaging agents used for inflammation imaging

Nuclear imaging modalities, such as single photon emission computed tomography (SPECT) and PET, offer functional and molecular information with high sensitivity. Of these two imaging modalities, PET is more sensitive and allows for quantitative imaging in a clinical setting [3]. The combination of molecular/functional and anatomic imaging is preferred in order to identify the exact location of the functional SPECT/PET signal.

Most of the imaging agents for inflammation imaging are SPECT imaging agents. Currently, the golden standard in nuclear medicine imaging of inflammation is autologous radiolabeled leukocytes [1,2]. They accumulate in the inflamed tissue by specific leukocyte migration. First, the injected radiolabeled white blood cells (WBCs) adhere to the endothelium and further migrate into the tissue. The leukocytes are labeled either with ^99m^Tc or ^111^In, with ^99m^Tc being the more commonly used radionuclide due to its nearly optimal radiation characteristics. However, the radiolabeling of white blood cells requires a skilled technician, is laborious, and involves the risk of handling contaminated blood. Also tissue-specific cells, such as inflammation-activated osteoblasts in the bone, can be detected with ^99m^Tc-labeled bisphosphonates. The characteristics of inflammation, such as increased blood flow and enhanced vascular permeability, can also be exploited in the imaging of inflammation. These nonspecific events can be imaged with ^99m^Tc-labeled nanocolloids or ^99m^Tc- and ^111^In-labeled proteins, such as immunoglobulin G (IgG) or albumin. In addition to these imaging agents, ^67^Ga-labeled citrate has also been used in the diagnosis of inflammatory diseases. ^67^Ga]citrate binds to transferrin, and this complex may extravasate to the sites of inflammation due to vascular permeability.

^18^F]FDG is used in inflammation imaging with high sensitivity [4,5]. The uptake of ^18^F]FDG is dependent on the metabolic rate of the cell and the number of glucose transporters. Since activated inflammatory cells show an increase in the number of glucose transporters, relative to control conditions, ^18^F]FDG could be used as a molecular tracer. In addition, PET, as a technique, allows for the quantitative measurements of ^18^F]FDG uptake, making it possible to evaluate the efficacy of treatment and progression of the disease. Several inflammatory/infectious conditions, such as fever of unknown origin, focal infections, osteomyelitis, and vasculitis, can be imaged with ^18^F]FDG [4,5]. Although ^18^F]FDG PET imaging shows potential in inflammation imaging, the uptake of ^18^F]FDG is not specific for inflammatory conditions.

### Adhesion molecules as targets for inflammation imaging

Targeting endothelial adhesion molecules may provide a more specific way to image inflammation through the detection of endothelial activation. Table 1 shows possible targets and available imaging agents for this purpose. Leukocyte migration from the blood into the non-lymphoid tissues is a hallmark of inflammation. Several molecules on the leukocyte surface and their counter-receptors on vascular endothelium mediate a multi-step adhesion cascade featuring tethering, rolling, activation, adhesion, crawling, and transmigration phases.

**Table 1:** Endothelial adhesion molecule candidates for the nuclear imaging and currently available imaging agents

**Target molecule**	**Imaging agent**	**Imaged condition**	**Reference**
E-selectin	^111^In- or ^99m^Tc-labeled anti-E-selectin mAb or fragments of it	Arthritis of pig, a pig model of gout, patients with RA, patients with IBD	[6-10]
E-selectin	^99m^Tc-labeled E-selectin-binding peptide	Rat model of arthritis	[11]
P-selectin	^99m^Tc-labeled F(Ab)2 fragment	Pulmonary embolism in beagle dog	[12]
VCAM-1	^123^I-labeled VCAM-1 antibody	Colitis in rat	[13]
VCAM-1	^123^I- and ^99m^Tc-labeled peptides	Atherosclerosis in rabbit, atherosclerotic plaques in mice	[14,15]
	^18^F-labeled tetrameric peptide		
ICAM-1	^111^In-labeled ICAM-1 antibody	Lung injury in rat	[16]
Integrin α_v_β_3_	^18^F-, ^64^Cu-, and ^68^Ga-labeled RGD peptides	Mouse model of chronic inflammation, acute and chronic model of inflammation in mice, atherosclerotic plaques in mice	[17-20]

### Imaging of selectins

Selectins are lectins that reach over the cell membrane and participate in the rolling step of the adhesion cascade. E- and P-selectin are expressed on inflamed endothelium in several human diseases, such as rheumatoid arthritis (RA) and inflammatory bowel disease (IBD). Preclinical studies with radiolabeled anti-E-selectin mAb confirmed the proof-of-principle of imaging for this endothelial adhesion molecule. Several animal models, such as the arthritis and gout model in pigs, proved that specific uptake of ^111^In-labeled anti-E-selectin mAb occurs in the inflamed tissue [6,7]. In addition to gout detection in pigs, Chapman and co-workers were able to follow the kinetics of E-selectin with a labeled antibody [7]. This ^111^In-labeled intact (murine) mAb was able to detect E-selectin on activated endothelium *in vivo* in patients with RA [8]. In comparison to human immunoglobulin G, an established imaging agent for RA, anti-E-selectin mAbs provided images with superior quality. In addition, labeled anti-E-selectin mAb showed good immunogenicity, since no human anti-mouse antibodies were detected at 12 to 14 days after mAb injection. Also, IBD in the patient was detected with ^111^In-labeled E-selectin Ab, and these results were comparable to radiolabeled WBCs [9]. Even though intact mAbs showed promise in E-selectin targeting and RA imaging, they are large molecules and suffer from slow blood clearance. To address this issue, a smaller fragment of the antibody was labeled with ^99m^Tc and used in the imaging of joint inflammation in patients [10]. ^99m^Tc-labeled Fab fragment of the mAb showed specific uptake as early as 4 h after the injection, in comparison to a nonspecific antibody. In addition to the antibody approach, a peptide labeled with ^99m^Tc shows promise in the imaging of inflamed synovium in a rat model of arthritis [11].

### Imaging of immunoglobulin superfamily members

The immunoglobulin superfamily is a family of cell surface receptors, which participate in many tasks, including antigen recognition and binding of the complement. Five of the members in this family participate in leukocyte adhesion, interacting mainly with integrins. Vascular cell adhesion molecule-1 (VCAM-1) and intercellular adhesion molecule 1 (ICAM-1) mediate arrest in the leukocyte extravasation process and are found in the vasculature in many inflammatory conditions. To date, only preclinical studies exist regarding imaging agents targeting VCAM-1 and ICAM-1. ^111^In-labeled antibody against ICAM-1 is specifically able to detect bleomycin-induced lung injury in rats as early as 4 h after induction [16]. Several animal models, such as the experimental model of colitis in rats, atherosclerosis in the rabbit model and atherosclerotic plaques, in mice, have been evaluated with VCAM-1 targeting imaging agents [13-15].

### Imaging of integrins

Integrins are a large member of adhesion molecules. The most extensively studied member of the integrin family is the integrin α_v_β_3_. Integrin α_v_β_3_ is a molecule which mediates the migration of endothelial cells through the basement membrane during blood vessel formation. This integrin is significantly upregulated on activated endothelial cells during angiogenesis, but not on inactive endothelial cells. This integrin serves as a molecular marker for tumor angiogenesis and metastasis imaging. In addition to cancer angiogenesis, integrin α_v_β_3_ expression is also found during wound healing, RA, psoriatic plaques, and during restenosis. Noninvasive monitoring of the molecular processes during angiogenesis would supply helpful information for clinicians, as well as for basic scientists. The most successful imaging agents for PET and SPECT imaging of integrin α_v_β_3_ are described [21-23].

Although, all clinical imaging studies with arginine-glycine-aspartic acid (RGD)-peptides concern cancer patients, imaging of α_v_β_3_ integrin may become a new biomarker for disease activity in inflammatory processes. Integrin α_v_β_3_ expression exists in many inflammatory diseases, such as RA. In imaging studies, only preclinical studies have exploited this opportunity. The radiolabeled RGD peptide accumulates in chronically inflamed tissue in mice due to of α_v_β_3_-specific binding, and this process can be monitored noninvasively with PET [17]. Also, the chronic phase of inflammation showed a higher uptake in comparison to acute inflammation evaluated by the ^64^Cu-labeled tetrameric RGD peptide [18]. In addition to these chemically induced inflammatory models, radiolabeled RGD peptides show specific accumulation in atherosclerotic plaques in genetically modified mice, which spontaneously develop atherosclerotic lesions [19,20].

### Vascular adhesion protein-1

Vascular adhesion protein-1 (VAP-1/SSAO) is an endothelial cell adhesion molecule that is rapidly translocated from the intracellular storage granules to the endothelial cell surface upon inflammation. VAP-1 participates in the recruitment of leukocytes to the sites of inflammation. It plays a role during the rolling, adhesion, and transmigration steps in the extravasation cascade mediating the journey of a leukocyte from the blood into the tissue [24]. The early translocation of VAP-1 makes it an optimal candidate for anti-inflammatory therapy and a potential target for the *in vivo* imaging of inflammation.

VAP-1 is a membrane-bound homodimer with the molecular weight of approximately 90 kDa per monomer [25]. Unlike the conventional homing-associated endothelial molecules, VAP-1 is also an enzyme. It degrades primary amines and produces hydrogen peroxide, ammonium and aldehyde as its enzymatic end products - all potent inflammatory mediates. Dietary methylamine is one of its substrate, but it can also use one of its leukocyte ligands, Siglec-10 as a substrate [26]. The crystal structure of VAP-1 is known [27]. The molecule has a heart-shaped fold that contains a transmembrane domain and extracellular domains. The D4 domain includes the enzymatically active site buried deep in the globular head of the molecule. The heart of the active site is the topaquinone that is the co-factor necessary for enzymatic activity. The active site is large enough to accommodate an amino acid side chain and might interact with a larger molecule, such as a peptide or protein ligand. VAP-1 contains several sites for glycosylation. Glycosylation is necessary for VAP-1 to function properly during leukocyte adhesion.

The current working hypothesis for leukocyte binding to VAP-1 describes this binding as having a dual mode of function, as described by Salmi and Jalkanen [28]. The leukocyte first makes contact with VAP-1 expressed on the endothelial cells by using its counter receptor to interact with the surface of VAP-1. This part is played by the VAP-1 epitope(s) recognized by certain monoclonal antibodies. After this initial contact, an amine in the surface of the leukocyte binds into the substrate channel of VAP-1. This leads to an enzymatic reaction between the leukocyte ligand (substrate) and the endothelial VAP-1. These two events are connected because the enzymatic function is needed for leukocyte binding [29]. Blocking of the antibody binding site with anti-VAP-1 monoclonal antibodies, as well as the inhibition of the enzymatic activity, reduces the number of adherent cells under flow conditions [30].

Even though VAP-1 plays an important role in the early events of inflammation, the expression of VAP-1 on the cell surfaces stays constant for a longer time periods, if the inflammation continues. This suggests that VAP-1 can still be targeted after the first phase of inflammation and makes it a promising target for anti-adhesive therapy [31]. Several animal models exist to obtain knowledge of the role of VAP-1 in human pathologies. For example, VAP-1 mediates lymphocyte binding to the vascular endothelium in rejected kidney allografts as well as in the liver allograft in rats [32,33]. VAP-1 also plays a role in neurological diseases such as forebrain ischemia and experimental autoimmune encephalitis, which is a rodent model for multiple sclerosis [34,35]. VAP-1 knockout and transgenic mice have confirmed the role of VAP-1 as an inflammatory adhesion molecule - knowledge that was originally obtained from inhibitory studies with antibodies [30,36].

### VAP-1 as a target for imaging

Imaging agents targeting VAP-1 could be valuable not only for the diagnosis and planning of treatment for patients, but also for the drug discovery and development processes. In addition to therapy monitoring, VAP-1 imaging would provide a scientific tool for *in vivo* imaging of leukocyte trafficking at the sites of inflammation. Several publications have appeared, to date, concerning VAP-1 in imaging (Table 2).

**Table 2:** Nuclear medicine imaging studies with VAP-1 targeting agents

**Imaging agent**	**Imaged condition**	**Reference**
^123^I-labeled anti-human-VAP-1 mAb 1B2	Animal models of skin inflammation and aseptic arthritis	[31]
^68^Ga-labeled VAP-1 binding peptide, ^68^Ga-DOTAVAP-P1	Animal model of osteomyelitis	[37,38]
^68^Ga-labeled VAP-1 binding peptide, ^68^Ga-DOTAVAP-P1	Animal model of sterile skin inflammation and pancreatic adenocarcinoma xenograft in rat	[39]
^68^Ga-labeled VAP-1 binding peptide, ^68^Ga-DOTAVAP-PEG-P1	Animal model of sterile skin inflammation in rat	[40]
^68^Ga-labeled VAP-1 binding peptide, ^68^Ga-DOTA-Siglec-9 peptide	Animal model of sterile skin inflammation in rat, DNBC-induced skin inflammation in hVAP-1 transgenic mouse and VAP-1 knockout mouse	[41]

The first publication to show the potential of VAP-1 as a target for *in vivo* imaging was conducted by Jaakkola and co-workers [31]. Anti-human-VAP-1 mAb 1B2 was labeled with ^123^I using the chloramine T method and evaluated in animal models of skin inflammation and aseptic arthritis. Inflammation was visualized and the uptake was specific in comparison to a simultaneously injected nonspecific antibody.

Exploiting the knowledge of the crystal structure of VAP-1, several small peptides were modeled to fit to the enzymatic groove of VAP-1. A specific lysine-containing peptide was found to be the inhibitor of the enzymatic activity of VAP-1 [42]. The peptide, GGGGKGGGG, was tested in functional binding assays and it effectively inhibited lymphocyte-endothelial-cell interaction under conditions of flow. This peptide was labeled with ^68^Ga and further evaluated as an imaging agent for PET imaging [37,39]. The preclinical testing of the VAP-1 peptide, ^68^Ga-DOTAVAP-P1, was positive when assessing *in vivo* stability, tissue distribution, and biokinetics. *In vivo* specificity exists in an animal model of osteomyelitis in comparison to negative control peptides, modeled to fit poorly in the enzymatic loop of VAP-1, and to two competitors (semicarbazide and an unlabeled peptide (DOTAVAP-P1). The study revealed a significantly lower uptake in the infected site for all negative controls. In comparison to ^18^F]FDG, ^68^Ga-DOTAVAP-P1 showed a slightly lower target-to-background ratio in a rat model of osteomyelitis. ^68^Ga-DOTAVAP-P1 was also able to accurately demonstrate the phase of inflammation in healing bones and the progress of a *Staphylococcus aureus* bacterial infection in osteomyelitic bones [37]. The uptake of ^68^Ga-DOTAVAP-P1 at the site of inflammation in an experimental sterile skin inflammation model was comparable to ^18^F]FDG. The results of ^68^Ga-DOTAVAP-P1 were in line with the expression of luminal VAP-1, which was high at the site of inflammation [39]. In all of these studies, intravenously administered ^68^Ga-DOTAVAP-P1 exhibited rapid excretion through the kidneys to the urinary bladder. Notable retention in the liver and very low radioactivity in the brain was observed. The high hydrophilicity of ^68^Ga-DOTAVAP-P1 (logD, −4.5) gives an explanation for the rapid renal excretion [38].

In the next study, ^68^Ga-DOTAVAP-P1 was chemically modified by adding a PEG linker to improve its pharmacokinetic properties [40]. The incorporation of a mini-PEG spacer in ^68^Ga-DOTAVAP-P1 enhanced its *in vivo* stability and improved PET imaging of the inflammation. This modification had no apparent effect on the *in vitro* properties of the VAP-1 binding peptide. Both peptides were stable in plasma incubations *in vitro* and their solubility was very similar. However, when intravenously administered, ^68^Ga-DOTAVAP-PEG-P1 showed significantly longer metabolic and elimination half-lives and slower total clearance compared to ^68^Ga-DOTAVAP-P1. Furthermore, our results revealed that while both peptides were able to detect experimental inflammation by PET imaging, ^68^Ga-DOTAVAP-PEG-P1 showed a higher inflammation-to-muscle ratio than the original ^68^Ga-DOTAVAP-P1. The renal excretion of ^68^Ga-DOTAVAP-PEG-P1 was slower, resulting in significantly lower urinary bladder radioactivity in comparison to ^68^Ga-DOTAVAP-P1.

Recently, a phage-display method discovered a novel VAP-1 binding peptide [41]. When further studies were conducted, this peptide was revealed to belong to an unknown leukocyte ligand of VAP-1. This peptide was part of a Siglec-9 molecule that is found on granulocytes and macrophages. The combination of VAP-1 as a target and its natural binding structure as an imaging tool provides a theoretically optimal setting. This peptide was labeled with ^68^Ga and used in PET imaging studies of experimental inflammation. Transgenic mice expressing human VAP-1 on endothelium showed a higher signal in chemically induced ear inflammation, in comparison to the control site, and to VAP-1 deficient knockout mice. The labeled peptide showed preferred renal excretion and some uptake in the liver.

**Figure 1:**
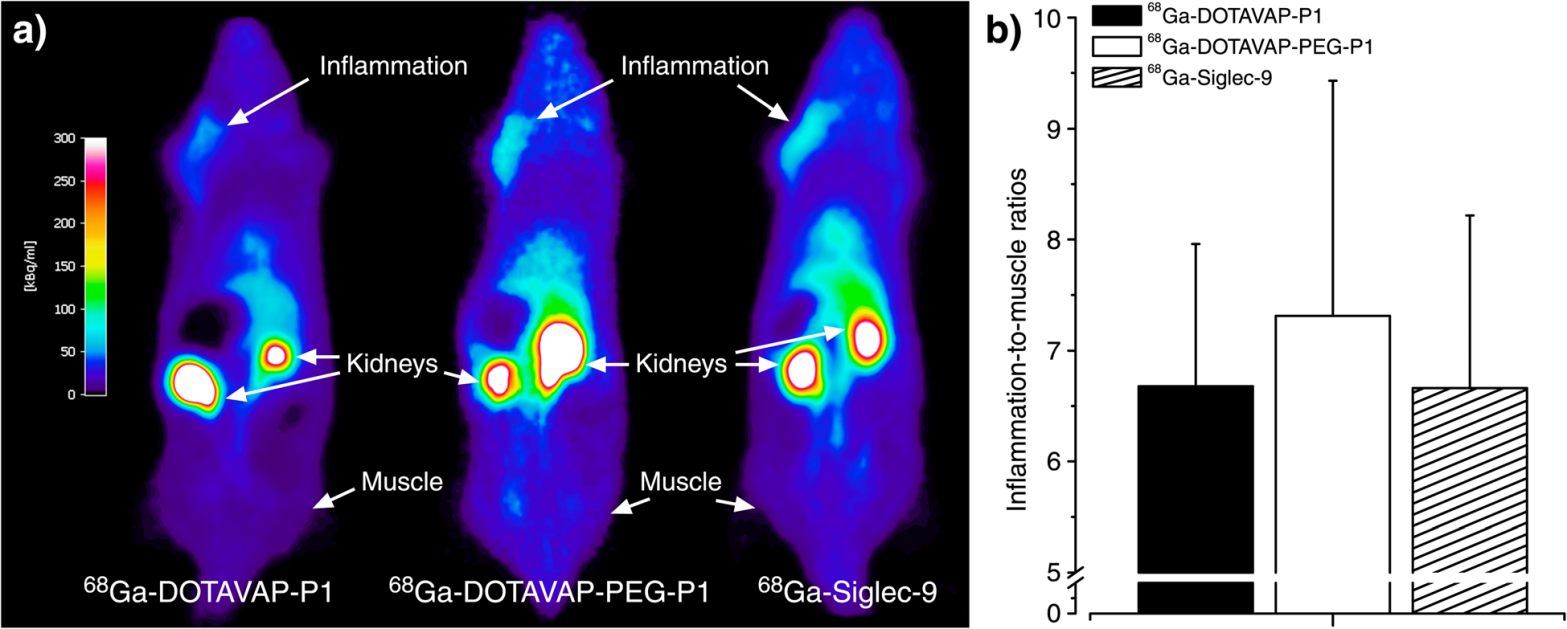
**VAP-1 targeting imaging agents in detecting inflammation.** (**a**) PET images of ^68^Ga-labeled VAP-1 binding peptides in a rat model of sterile skin/muscle inflammation. All the images fall within the same radioactivity scale and the scale bar is shown on the left. (**b**) Inflammation-to-muscle ratios obtained from PET imaging at 60 min after injection.

All of these peptides were tested using the same turpentine-induced rat model of sterile inflammation, and the results can thus be compared. All of these peptides were able to detect inflammation from the background muscle in this model, as seen in Figure 1. Peptides had high target-to-non target ratios (above 6) and showed rapid uptake in the inflammatory foci as well as preferred renal clearance and some uptake in the liver. The standardized uptake values derived from PET images were 0.33 ± 0.07, 0.53 ± 0.01, and 0.45 ± 0.06 for ^68^Ga-DOTAVAP-P1, ^68^Ga-DOTAVAP-PEG-P1, and ^68^Ga-DOTA-Siglec-9 peptide, respectively, at 60 min after injection.

Due to its novel characteristics among various homing-associated molecules, VAP-1 has certain benefits as a target for imaging inflammation. Unlike ICAM-1, VCAM-1, and integrin α_v_β_3_, it is hardly detected on the endothelium in normal conditions, thus theoretically providing better specificity to detect inflammation. Moreover, its enzymatic activity provides additional ways to design imaging targets in comparison to endothelial selectins.

### Limitations in VAP-1 imaging

VAP-1 binding peptides and antibodies have few limitations in PET imaging. The imaging of the abdomen area is limited, since the liver shows high uptake. The liver uptake is, at least in part, due to the high number of VAP-1 receptors in the sinusoidal endothelial cells in the human liver [43]. Some degradation products of ^68^Ga-DOTA-peptides, such as free ^68^Ga, also tend to accumulate in the liver [44]. In studies with ^68^Ga-labeled Siglec-9 peptide and blocking and a comparison of VAP-1 positive WT mice and VAP-1 negative KO mice revealed 60% and 50% specific binding of the peptide in the livers of each genotype of mouse, respectively [41]. Since VAP-1 has a soluble form in the circulation, which is enzymatically cleaved from the endothelial cell surface [30], the binding of labeled peptides to soluble VAP-1 might theoretically limit their use as an imaging target in cases in which the soluble VAP-1 concentrations are very high. In our preclinical studies, all inflammatory and cancer focuses were detectable from the background tissue, indicating that the soluble form does not present an issue. In addition, the excretion to the urine via the kidneys limits the use of these peptides for imaging of the pelvic area.

A tracer capable of distinguishing between microbial infection and sterile inflammation would have clinical value. Unfortunately, VAP-1 targeting agents are not probably able to do this, since VAP-1 is expressed, at least in some bacterial infections, as well as in the sterile inflammation [37]. However, the inclusion of kinetic PET data will likely give rise to a better quantification of VAP-1 expression in local tissues.

### Future prospects

VAP-1 is upregulated in many human inflammatory diseases, such as acute myocardial infarction, inflammatory bowel disease and rheumatoid arthritis [45-48]; and a fully humanized anti-VAP-1 antibody is used in clinical trials for patients suffering from rheumatoid arthritis and psoriasis [49]. Leukocyte extravasation from the blood into inflamed tissues plays a crucial role in the pathogenesis of these diseases and therefore, molecular imaging of the adhesion molecules expressed on the vasculature of the target organs, in these diseases, could help in the diagnosis and evaluation of the severity of these diseases. Moreover, successful imaging could be beneficial in the selection of patients for specific antibody treatment and in monitoring of these treatments and disease progression. This approach could also be utilized in VAP-1 specific drug development and the selection of patients for clinical trials.

## Conclusions

During recent years, several publications reporting the use of homing-associated endothelial cell molecules as targets to image inflammation emerged. As being important in leukocyte trafficking to the sites of inflammation, they are logical targets for developing novel agents for imaging. Over time, a molecular targeting strategy is likely to prevail over the existing white cell labeling technique. One of the most promising candidates among the homing-associated molecules is VAP-1 due to its unique characteristics in being both an adhesion and enzymatic molecule. Moreover, its rapid translocation to the endothelial cell surface at the sites of inflammation increases its potential as an imaging target. Several preclinical studies of VAP-1 targeting with PET imaging agents exist, and successful detection of VAP-1 in the vasculature is described. These results strongly suggest that VAP-1 could be used as a target in the diagnosis of various inflammatory conditions also in humans. Future studies will show how well these expectations are fulfilled.

## Abbreviations

IBD: Inflammatory bowel disease; ICAM-1: Intercellular adhesion molecule 1; RA: Rheumatoid arthritis; SSAO: Semicarbazide sensitive amine oxidase; VAP-1: Vascular adhesion protein-1; VCAM-1: Vascular cell adhesion molecule-1; WBC: White blood cells.

## Competing interest

The authors declare that they have no competing interests.

## Authors’ contributions

AA reviewed current literature and wrote the manuscript. AR provided valuable intellectual advice on the imaging studies. SJ provided valuable input to the biology of the target molecule VAP-1. AR and SJ critically reviewed the manuscript. All the authors read and approved the final manuscript.

## References

[B1] Boerman OC Rennen H Oyen WJ Corstens FH Radiopharmaceuticals to image infection and inflammation Semi Nucl Med 2001 31 286 295 10.1053/snuc.2001.26189 11710771

[B2] Gotthardt M Bleeker-Rovers CP Boerman OC Oyen WJ Imaging of inflammation by PET, conventional scintigraphy, and other imaging techniques J Nucl Med 2010 51 1937 1949 10.2967/jnumed.110.076232 21078798

[B3] Rahmim A Zaidi H PET versus SPECT: strengths, limitations and challenges Nucl Med Commun 2008 29 193 207 10.1097/MNM.0b013e3282f3a515 18349789

[B4] Love C Tomas MB Tronco GG Palestro CJ FDG PET of infection and inflammation Radiographics 2005 25 1357 1368 10.1148/rg.255045122 16160116

[B5] Vos FJ Bleeker-Rovers CP Corstens FH Kullberg BJ Oyen WJ FDG-PET for imaging of non-osseous infection and inflammation Q J Nucl Med Mol Imaging 2006 50 121 130 16770302

[B6] Keelan ET Harrison AA Chapman PT Binns RM Peters AM Haskard DO Imaging vascular endothelial activation: an approach using radiolabeled monoclonal antibodies against the endothelial cell adhesion molecule E-selectin J Nucl Med 1994 35 276 281 7507524

[B7] Chapman PT Jamar F Harrison AA Schofield JB Peters AM Binns RM Haskard DO Characterization of E-selectin expression, leucocyte traffic and clinical sequelae in urate crystal-induced inflammation: an insight into gout Br J Rheumatol 1996 35 323 334 10.1093/rheumatology/35.4.323 8624635

[B8] Chapman PT Jamar F Keelan ET Peters AM Haskard DO Use of a radiolabeled monoclonal antibody against E-selectin for imaging of endothelial activation in rheumatoid arthritis Arthritis Rheum 1996 39 1371 1375 10.1002/art.1780390815 8702446

[B9] Bhatti M Chapman P Peters M Haskard D Hodgson HJ Visualising E-selectin in the detection and evaluation of inflammatory bowel disease Gut 1998 43 40 47 10.1136/gut.43.1.40 9771404 PMC1727171

[B10] Jamar F Houssiau FA Devogelaer JP Chapman PT Haskard DO Beaujean V Beckers C Manicourt DH Peters AM Scintigraphy using a technetium 99m-labelled anti-E-selectin Fab fragment in rheumatoid arthritis Rheumatology 2002 41 53 61 10.1093/rheumatology/41.1.53 11792880

[B11] Zinn KR Chaudhuri TR Smyth CA Wu Q Liu HG Fleck M Mountz JD Mountz JM Specific targeting of activated endothelium in rat adjuvant arthritis with a 99mTc-radiolabeled E-selectin-binding peptide Arthritis Rheum 1999 42 641 649 10.1002/1529-0131(199904)42:4<641::AID-ANR6>3.0.CO;2-T 10211877

[B12] Ji S Fang W Zhu M Bai X Wang C Ruan C Detection of pulmonary embolism with 99mTc-labeled F(ab)2 fragment of anti-P-selectin monoclonal antibody in dogs Tohoku J Exp Med 2011 223 9 15 10.1620/tjem.223.9 21187695

[B13] Sans M Fuster D Vázquez A Setoain FJ Piera C Piqué JM Panés J 123Iodine-labelled anti-VCAM-1 antibody scintigraphy in the assessment of experimental colitis Eur J Gastroenterol Hepatol 2001 13 31 38 10.1097/00042737-200101000-00006 11204806

[B14] Broisat A Riou LM Ardisson V Boturyn D Dumy P Fagret D Ghezzi C Molecular imaging of vascular cell adhesion molecule-1 expression in experimental atherosclerotic plaques with radiolabelled B2702-p Eur J Nucl Med Mol Imaging 2007 34 830 840 10.1007/s00259-006-0310-4 17219135

[B15] Nahrendorf M Keliher E Panizzi P Zhang H Hembrador S Figueiredo JL Aikawa E Kelly K Libby P Weissleder R 18F-4V for PET-CT imaging of VCAM-1 expression in atherosclerosis JACC Cardiovasc Imaging 2009 2 1213 1222 10.1016/j.jcmg.2009.04.016 19833312 PMC2773129

[B16] Weiner RE Sasso DE Gionfriddo MA Syrbu SI Smilowitz HM Vento J Thrall RS Early detection of bleomycin-induced lung injury in rat using indium-111-labeled antibody directed against intercellular adhesion molecule-1 J Nucl Med 1998 39 723 728 9544689

[B17] Pichler BJ Kneilling M Haubner R Braumüller H Schwaiger M Röcken M Weber WA Imaging of delayed-type hypersensitivity reaction by PET and 18F-galacto-RGD J Nucl Med 2005 46 184 189 15632051

[B18] Cao Q Cai W Li ZB Chen K He L Li HC Hui M Chen X PET imaging of acute and chronic inflammation in living mice Eur J Nucl Med Mol Imaging 2007 34 1832 1842 10.1007/s00259-007-0451-0 17541586

[B19] Laitinen I Saraste A Weidl E Poethko T Weber AW Nekolla SG Leppänen P Ylä-Herttuala S Hölzlwimmer G Walch A Esposito I Wester HJ Knuuti J Schwaiger M Evaluation of alphavbeta3 integrin-targeted positron emission tomography tracer 18F-galacto-RGD for imaging of vascular inflammation in atherosclerotic mice Circ Cardiovasc Imaging 2009 2 331 338 10.1161/CIRCIMAGING.108.846865 19808614

[B20] Haukkala J Laitinen I Luoto P Iveson P Wilson I Karlsen H Cuthbertson A Laine J Leppänen P Ylä-Herttula S Knuuti J Roivainen A 68Ga-DOTA-RGD peptide: biodistribution and binding into atherosclerotic plaques in mice Eur J Nucl Med Mol Imaging 2009 36 2058 2067 10.1007/s00259-009-1220-z 19629477

[B21] Cai W Gambhir SS Chen X Multimodality tumor imaging targeting integrin alphavbeta3 Biotechniques 2005 39 Suppl 14 25 10.2144/000112091 20158499

[B22] Haubner R Alphavbeta3-integrin imaging: a new approach to characterise angiogenesis? Eur J Nucl Med Mol Imaging 2006 33 Suppl 54 63 16791598 10.1007/s00259-006-0136-0

[B23] Beer AJ Schwaiger M Imaging of integrin alphavbeta3 expression Cancer Metastasis Rev 2008 27 631 644 10.1007/s10555-008-9158-3 18523730

[B24] Salmi M Jalkanen S VAP-1: an adhesin and an enzyme Trends Immunol 2001 22 211 216 10.1016/S1471-4906(01)01870-1 11274927

[B25] Salmi M Jalkanen S Human vascular adhesion protein 1 (VAP-1) is a unique sialoglycoprotein that mediates carbohydrate-dependent binding of lymphocytes to endothelial cells J Exp Med 1996 183 569 579 10.1084/jem.183.2.569 8627168 PMC2192471

[B26] Kivi E Elima K Aalto K Nymalm Y Auvinen K Koivunen E Otto DM Crocker PR Salminen TA Salmi M Jalkanen S Human Siglec-10 can bind to vascular adhesion protein-1 and serves as its substrate Blood 2009 114 5385 5392 10.1182/blood-2009-04-219253 19861682 PMC2978503

[B27] Airenne TT Nymalm Y Kidron H Smith DJ Pihlavisto M Salmi M Jalkanen S Johnson MS Salminen TA Crystal structure of the human vascular adhesion protein-1: unique structural features with functional implications Protein Sc 2005 14 1964 1974 10.1110/ps.051438105 16046623 PMC2279308

[B28] Salmi M Jalkanen S Homing-associated molecules CD73 and VAP-1 as targets to prevent harmful inflammations and cancer spread FEBS Lett 2011 585 1543 1550 10.1016/j.febslet.2011.04.033 21515268

[B29] Salmi M Yegutkin GG Lehvonen R Koskinen K Salminen T Jalkanen S A cell surface amine oxidase directly controls lymphocyte migration Immunity 2001 14 265 276 10.1016/S1074-7613(01)00108-X 11290336

[B30] Stolen CM Yegutkin GG Kurkijärvi R Bono P Alitalo K Jalkanen S Origins of serum semicarbazide-sensitive amine oxidase Circ Res 2004 95 50 57 10.1161/01.RES.0000134630.68877.2F 15178639

[B31] Jaakkola K Nikula T Holopainen R Vähäsilta T Matikainen MT Laukkanen ML Huupponen R Halkola L Nieminen L Hiltunen J Parviainen S Clark MR Knuuti J Savunen T Kääpä P Voipio-Pulkki LM Jalkanen S In vivo detection of vascular adhesion protein-1 in experimental inflammation Am J Pathol 2000 157 463 471 10.1016/S0002-9440(10)64558-0 10934150 PMC1850117

[B32] Kurkijärvi R Jalkanen S Isoniemi H Salmi M Vascular adhesion protein-1 (VAP-1) mediates lymphocyte-endothelial interactions in chronic kidney rejection Eur J Immunol 2001 31 2876 2884 10.1002/1521-4141(2001010)31:10<2876::AID-IMMU2876>3.0.CO;2-Z 11592062

[B33] Martelius T Salaspuro V Salmi M Krogerus L Höckerstedt K Jalkanen S Lautenschlager I Blockade of vascular adhesion protein-1 inhibits lymphocyte infiltration in rat liver allograft rejection Am J Pathol 2004 165 1993 2001 10.1016/S0002-9440(10)63250-6 15579442 PMC1618725

[B34] Xu HL Salter-Cid L Linnik MD Wang EY Paisansathan C Pelligrino DA Vascular adhesion protein-1 plays an important role in postischemic inflammation and neuropathology in diabetic, estrogen-treated ovariectomized female rats subjected to transient forebrain ischemia J Pharmacol Exp Ther 2006 317 19 29 16339390 10.1124/jpet.105.096958

[B35] O'Rourke AM Wang EY Salter-Cid L Huang L Miller A Podar E Gao HF Jones DS Linnik MD Benefit of inhibiting SSAO in relapsing experimental autoimmune encephalomyelitis J Neural Transm 2007 114 845 849 10.1007/s00702-007-0699-3 17393060

[B36] Stolen CM Marttila-Ichihara F Koskinen K Yegutkin GG Turja R Bono P Skurnik M Hänninen A Jalkanen S Salmi M Absence of the endothelial oxidase AOC3 leads to abnormal leukocyte traffic in vivo Immunity 2005 22 105 115 10.1016/j.immuni.2004.12.006 15664163

[B37] Lankinen P Mäkinen TJ Pöyhönen TA Virsu P Salomäki S Hakanen AJ Jalkanen S Aro HT Roivainen A (68)Ga-DOTAVAP-P1 PET imaging capable of demonstrating the phase of inflammation in healing bones and the progress of infection in osteomyelitic bones Eur J Nucl Med Mol Imaging 2008 35 352 364 10.1007/s00259-007-0637-5 18038133

[B38] Ujula T Salomäki S Virsu P Lankinen P Mäkinen TJ Autio A Yegutkin GG Knuuti J Jalkanen S Roivainen A Synthesis, 68Ga labeling and preliminary evaluation of DOTA peptide binding vascular adhesion protein-1: a potential PET imaging agent for diagnosing osteomyelitis Nucl Med Biol 2009 36 631 641 10.1016/j.nucmedbio.2009.04.008 19647169

[B39] Autio A Ujula T Luoto P Salomäki S Jalkanen S Roivainen A PET imaging of inflammation and adenocarcinoma xenografts using vascular adhesion protein 1 targeting peptide [68Ga]-DOTAVAP-P1: comparison with [18F]-FDG Eur J Nucl Med Mol Imaging 2010 37 1918 1925 10.1007/s00259-010-1497-y 20523988

[B40] Autio A Henttinen T Sipilä HJ Jalkanen S Roivainen A Mini-PEG spacering of VAP-1-targeting [68Ga]-DOTAVAP-P1 peptide improves PET imaging of inflammation EJNMMI Research 2011 1 10 10.1186/2191-219X-1-10 22214508 PMC3251254

[B41] Aalto K Autio A Kiss EA Elima K Nymalm Y Veres TZ Marttila-Ichihara F Elovaara H Saanijoki T Crocker PR Maksimow M Bligt E Salminen TA Salmi M Roivainen A Jalkanen S Siglec-9 is a novel leukocyte ligand for vascular adhesion protein-1 and can be used in PET imaging of inflammation and cancer Blood 2011 118 3725 3733 10.1182/blood-2010-09-311076 21821708 PMC3833035

[B42] Yegutkin GG Salminen T Koskinen K Kurtis C McPherson MJ Jalkanen S Salmi M A peptide inhibitor of vascular adhesion protein-1 (VAP-1) blocks leukocyte-endothelium interactions under shear stress Eur J Immunol 2004 34 2276 2285 10.1002/eji.200424932 15259025

[B43] Lalor PF Edwards S McNab G Salmi M Jalkanen S Adams DH Vascular adhesion protein-1 mediates adhesion and transmigration of lymphocytes on human hepatic endothelial cells J Immunol 2002 169 983 992 12097405 10.4049/jimmunol.169.2.983

[B44] Ujula T Salomäki S Autio A Luoto P Tolvanen T Lehikoinen P Viljanen T Sipilä H Härkönen P Roivainen A 68Ga-chloride PET reveals human pancreatic adenocarcinoma xenografts in rats–comparison with FDG Mol Imaging Biol 2010 12 259 268 10.1007/s11307-009-0267-3 19798536 PMC2864902

[B45] Salmi M Jalkanen S A 90-kilodalton endothelial cell molecule mediating lymphocyte binding in humans Science 1992 257 1407 1409 10.1126/science.1529341 1529341

[B46] Salmi M Kalimo K Jalkanen S Induction and function of vascular adhesion protein-1 at sites of inflammation J Exp Med 1993 178 2255 2260 10.1084/jem.178.6.2255 8245796 PMC2191278

[B47] Salmi M Rajala P Jalkanen S Homing of mucosal leukocytes to joints. Distinct endothelial ligands in synovium mediate leukocyte-subtype specific adhesion J Clin Invest 1997 99 2165 2172 10.1172/JCI119389 9151788 PMC508046

[B48] Jaakkola K Jalkanen S Kaunismäki K Vänttinen E Saukko P Alanen K Kallajoki M Voipio-Pulkki LM Salmi M Vascular adhesion protein-1, intercellular adhesion molecule-1 and P-selectin mediate leukocyte binding to ischemic heart in humans J Am Coll Cardiol 2000 36 122 129 10.1016/S0735-1097(00)00706-3 10898423

[B49] Biotie Therapieshttp://www.biotie.com/en/product_and_development/inflammation/vap1_antibody. Accessed 23 August 2012

